# Supplementary material for: Overcoming false-positive gene-category enrichment in the analysis of spatially resolved transcriptomic brain atlas data
Source: Nat Commun. 2021 May 11;12:2669. doi: 10.1038/s41467-021-22862-1 (PMC8113439; doi:10.1038/s41467-021-22862-1)
Supplement: Supplementary file 3 — Description of Additional Supplementary Files [file 41467_2021_22862_MOESM3_ESM.docx]

**Description of Additional Supplementary Files**

**Title: Supplementary Data 1**

**Description:** A table of GO Categories annotated by the number of mouse GCEA studies and the number of human GCEA studies from our literature review that have reported a significant association (*p* < 0.05) with a spatial brain phenotype. Labels to specific analyses that implicate each GO category are also annotated (see code repository for details).

**Title: Supplementary Data 2**

**Description:** Table containing computed GO category false-positive rates (CFPRs) in mouse and human. Results are provided for the SBP-random ensemble (Columns: ‘CFPR_Mouse_SBPrandom’ and ‘CFPR_Human_SBPrandom’) and the SBP-spatial null ensemble (Columns: ‘CFPR_Mouse_SBPspatial’ and ‘CFPR_Human_SBPspatial’), as well as computed reference CFPRs (Columns: ‘CFPR_Mouse_Reference’ and ‘CFPR_Human_Reference’). Also included is a count of the number of published analyses from our literature survey that have reported a significant association with each GO category, in mouse (Column: ‘mouseLiterature’) and human (Column: ‘humanLiterature’).

**Title: Supplementary Data 3**

**Description:** A table containing within-category coexpression scores, <*r*>, for all GO categories in mouse and human.

**Title: Supplementary Data 4**

**Description:** Excel file containing fitted spatial autocorrelation parameters for all GO categories in human cortex and mouse brain (across two Sheets, as labeled). For each GO category, the tables list parameters estimated from the exponential fit: *A*, *B*, *λ*, *R*_exp_^2^, and the model-free Spearman correlation with CGE, –*ρ* (see Methods for definitions).
