# Supplementary material for: Overcoming false-positive gene-category enrichment in the analysis of spatially resolved transcriptomic brain atlas data
Source: Nat Commun. 2021 May 11;12:2669. doi: 10.1038/s41467-021-22862-1 (PMC8113439; doi:10.1038/s41467-021-22862-1)
Supplement: Supplementary file 8 — Reporting Summary [file 41467_2021_22862_MOESM8_ESM.pdf]

nature research

Corresponding author(s): Fulcher

Last updated by author(s): May 12, 2020

Reporting Summary

Nature Research wishes to improve the reproducibility of the work that we publish. This form provides structure for consistency and transparency in reporting. For further information on Nature Research policies, see our Editorial Policies and the Editorial Policy Checklist.

Statistics

For all statistical analyses, confirm that the following items are present in the figure legend, table legend, main text, or Methods section.

n/a	Confirmed	
☒	☐	The exact sample size (*n*) for each experimental group/condition, given as a discrete number and unit of measurement
☒	☐	A statement on whether measurements were taken from distinct samples or whether the same sample was measured repeatedly
☐	☒	The statistical test(s) used AND whether they are one- or two-sided<br>*Only common tests should be described solely by name; describe more complex techniques in the Methods section.*
☒	☐	A description of all covariates tested
☐	☒	A description of any assumptions or corrections, such as tests of normality and adjustment for multiple comparisons
☐	☒	A full description of the statistical parameters including central tendency (e.g. means) or other basic estimates (e.g. regression coefficient) AND variation (e.g. standard deviation) or associated estimates of uncertainty (e.g. confidence intervals)
☐	☒	For null hypothesis testing, the test statistic (e.g. *F*, *t*, *r*) with confidence intervals, effect sizes, degrees of freedom and *P* value noted<br>*Give P values as exact values whenever suitable.*
☒	☐	For Bayesian analysis, information on the choice of priors and Markov chain Monte Carlo settings
☒	☐	For hierarchical and complex designs, identification of the appropriate level for tests and full reporting of outcomes
☐	☒	Estimates of effect sizes (e.g. Cohen's *d*, Pearson's *r*), indicating how they were calculated

Our web collection on statistics for biologists contains articles on many of the points above.

Software and code

Policy information about availability of computer code

Data collection	No new data were collected in this study. All data required to reproduce results in this study are in the Zenodo repositories http://doi.org/10.5281/zenodo.3777553 and https://doi.org/10.5281/zenodo.4460713.
Data analysis	Full instructions for reproducing every analysis presented in the paper is provided in the Github repository https://github.com/benfulcher/GCEA_FalsePositives. A toolbox for running our technique on new data is also provided at https://github.com/benfulcher/GeneCategoryEnrichmentAnalysis. Both repositories have been snapshotted on Zenodo with associated DOIs: https://doi.org/10.5281/zenodo.4470239 and https://doi.org/10.5281/zenodo.3923470.

For manuscripts utilizing custom algorithms or software that are central to the research but not yet described in published literature, software must be made available to editors and reviewers. We strongly encourage code deposition in a community repository (e.g. GitHub). See the Nature Research guidelines for submitting code & software for further information.

Data

Policy information about availability of data

All manuscripts must include a data availability statement. This statement should provide the following information, where applicable:

- Accession codes, unique identifiers, or web links for publicly available datasets
- A list of figures that have associated raw data
- A description of any restrictions on data availability

Please see manuscript for full Data availability statement.

Field-specific reporting

Please select the one below that is the best fit for your research. If you are not sure, read the appropriate sections before making your selection.

☒ Life sciences ☐ Behavioural & social sciences ☐ Ecological, evolutionary & environmental sciences

For a reference copy of the document with all sections, see nature.com/documents/nr-reporting-summary-flat.pdf

Life sciences study design

All studies must disclose on these points even when the disclosure is negative.

Sample size	This paper aimed to demonstrate statistical issues associated with performing standard enrichment analyses in the literature. As such, we used state of the art transcriptional atlas data as is currently used for these analyses—the Allen Human Brain Atlas and the Allen Mouse Brain Atlas. As such, sample size consderations were not relevant to this study.
Data exclusions	A small minority of genes with poor expression quality were excluded from our analyses according to quality-control criteria described in Methods.
Replication	Replication is not applicable to this study, as the study's aim was to characterize statistical issues with a common methodology, not make scientific claims that would be subject to replication.
Randomization	Randomization is not appropriate to this study as no new data were collected and our aim was to characterize a statistical methodology.
Blinding	Blinding is not appropriate to this study as no new data were collected and our aim was to characterize a statistical methodology.

Reporting for specific materials, systems and methods

We require information from authors about some types of materials, experimental systems and methods used in many studies. Here, indicate whether each material, system or method listed is relevant to your study. If you are not sure if a list item applies to your research, read the appropriate section before selecting a response.

Materials & experimental systems

n/a	Involved in the study	
☒	☐	Antibodies
☒	☐	Eukaryotic cell lines
☒	☐	Palaeontology and archaeology
☒	☐	Animals and other organisms
☒	☐	Human research participants
☒	☐	Clinical data
☒	☐	Dual use research of concern

Methods

n/a	Involved in the study	
☒	☐	ChIP-seq
☒	☐	Flow cytometry
☒	☐	MRI-based neuroimaging
